# Diversity amongst trigeminal neurons revealed by high throughput single cell sequencing

**DOI:** 10.1371/journal.pone.0185543

**Published:** 2017-09-28

**Authors:** Minh Q. Nguyen, Youmei Wu, Lauren S. Bonilla, Lars J. von Buchholtz, Nicholas J. P. Ryba

**Affiliations:** Taste and Smell Section, National Institute of Dental and Craniofacial Research, National Institutes of Health, Bethesda, Maryland, United States of America; Indiana University School of Medicine, UNITED STATES

## Abstract

The trigeminal ganglion contains somatosensory neurons that detect a range of thermal, mechanical and chemical cues and innervate unique sensory compartments in the head and neck including the eyes, nose, mouth, meninges and vibrissae. We used single-cell sequencing and in situ hybridization to examine the cellular diversity of the trigeminal ganglion in mice, defining thirteen clusters of neurons. We show that clusters are well conserved in dorsal root ganglia suggesting they represent distinct functional classes of somatosensory neurons and not specialization associated with their sensory targets. Notably, functionally important genes (e.g. the mechanosensory channel Piezo2 and the capsaicin gated ion channel Trpv1) segregate into multiple clusters and often are expressed in subsets of cells within a cluster. Therefore, the 13 genetically-defined classes are likely to be physiologically heterogeneous rather than highly parallel (i.e., redundant) lines of sensory input. Our analysis harnesses the power of single-cell sequencing to provide a unique platform for in silico expression profiling that complements other approaches linking gene-expression with function and exposes unexpected diversity in the somatosensory system.

## Introduction

Somatosensory neurons have cell-bodies in the trigeminal and dorsal root ganglia and respond to a diverse array of thermal, mechanical and chemical stimuli to generate a wide variety of distinct sensations and behavioral responses [1, 2]. These primary sensory neurons differ widely in the morphology of their peripheral and central projections, have a range of axon diameters and conduction velocities and exhibit diverse functional tuning [1, 2]. Many genes have been linked to the detection specificity and response properties of somatosensory neurons. For example, cells expressing the heat sensitive ion channel, Trpv1, are required for normal responses to high temperature [3, 4], whereas those expressing the cool-temperature activated channel, Trpm8, appear dedicated sensors of cold [4–7]. However, functional imaging experiments have hinted at far greater diversity even within these thermosensory populations [8]. For example, four classes of cooling responsive neurons (including one group that only respond after injury) were very recently identified in recordings from the trigeminal ganglion [8]. Does molecular diversity amongst Trpm8-neurons explain this level of functional variability?

Advances in DNA sequencing have made single cell transcriptome (sc-transcriptome) analysis a possibility and potentially transforming approach to address such questions [9]. Until recently, exploiting this technology to obtain sequence from more than a handful of cells was both arduous and very expensive. However, the development of microfluidic approaches like Dropseq [10], Indrop sequencing [11] and commercial platforms (e.g. 10x Genomics) transformed the field by greatly simplifying and reducing the cost of large scale sc-transcriptome analysis. These methods rely on microfluidic co-capture of cells and barcoded primers in nanoliter droplets to differentially label transcripts from thousands of cells. Such sequencing approaches and related combinatorial strategies for barcoding [12] appear ideally suited for exploring how the molecular profiles of neural populations are linked to their functional and morphological diversity. Indeed, Dropseq was initially used to analyze the neurons and receptor cells in the mouse retina, and when extended to sufficient cell numbers, unbiased clustering effectively identified 15 classes of bipolar neurons [13]. Notably, this analysis segregates all the bipolar cell classes that have been demonstrated by morphology, localization studies and painstaking recordings across many groups and years [14] but also identified two new types of bipolar neurons.

Like the retina, somatosensory ganglia provide a rich source of spatially restricted neurons that are particularly attractive for sc-transcriptome analysis. Over the past few years, several studies [15–17] have analyzed single cell transcriptomics of somatosensory neurons isolated from dorsal root ganglia (DRG). These data provide important insight into neural diversity in the DRG, most clearly identifying proprioceptive and itch responsive neurons on the basis of their highly distinct expression profiles. However, several other known functional classes of neurons are less well delineated by gene expression patterns, and, for example, cold responsive neurons (marked by Trpm8 expression) are sparsely represented and are not consistently separated from other types of cells [15–17].

Here, we focused on the trigeminal ganglion, which has several features that differ from DRG, including lack of proprioceptive neurons, differences in embryonic origin as well as innervation of unique sensory environments including the oral and nasal cavities, the eyes, meninges and vibrissae [18]. We used the Dropseq technique [10] to significantly expand the depth of cell coverage in somatosensory sc-transcriptome analysis relative to previous studies [15–17]. Our results demonstrate that unbiased clustering of trigeminal neurons identifies about a dozen stable groupings or neural clusters. Importantly, most of the groups that we identified can be assigned functional roles on the basis of their molecular expression patterns. For example, Trpm8 marks well separated clusters of trigeminal neurons that likely respond to cooling. Thus this trigeminal sc-transcriptome resource provides a valuable in silico approach for examining expression patterns of large numbers of genes and consequently a rapid way to assess the cellular and potential molecular network role of candidate proteins in somatosensory transduction.

## Materials and methods

### Mice and isolation of trigeminal neurons

Animal experiments were carried out in strict accordance with the US National Institutes of Health (NIH) guidelines for the care and use of laboratory animals. This study was approved by the Institutional Animal Care and Use Committee (NIDCR IACUC, protocol number 14–744). FVB/N mice (Harlan) were euthanized by CO_2_ inhalation followed by cervical dislocation for tissue harvesting. Briefly, mice were slowly exposed to 100% CO_2_ at a flow rate displacing 10–30% of the cage volume/minute, once mice were unconscious, death was confirmed by cervical dislocation. Trigeminal ganglia were isolated from 20–30 mice (3–5 weeks old) of both sexes for each Dropseq run. Trigeminal ganglia were incubated in ice-cold PBS on ice until tissues from all animals were harvested (all steps were performed as rapidly as possible with incubation on ice when higher temperature was not required). For cell isolation, tissue from up to 10 animals was combined for a single tube. Dissociation of trigeminal neurons was carried out using the postnatal neural tissue dissociation kit (Miltenyi Biotec) following the manufacturer’s instruction. The dissociated cells were passed through a 100 μm filter and centrifuged at 300 g. The pellet was resuspended in 1 ml of Dulbecco’s phosphate buffered saline (DPBS) supplemented with 0.01% bovine serum albumin (BSA), and was loaded onto a 12.5% / 20% Percoll gradient, and centrifuged at 1,300g for 10 minutes. The top layer (12.5% Percoll) and interface were discarded, 4 ml of 0.01% BSA supplemented DPBS were added to the lower layer to dilute Percoll to 10% and the sample was centrifuged for 6 minutes at 1,000 g. The resulting cell pellet was resuspended in 80 μl of DPBS-BSA. Glia and other non-neuronal cells were depleted using the mouse neuron isolation kit (Miltenyi Biotec), following the manufacturer’s instruction. After removal of non-neuronal cells, the resulting pellet was re-suspended in 0.5 ml PBS-BSA and the number of cells was determined by counting under phase contrast illumination and adjusted (see below) in preparation for a Dropseq run. We monitored the sample at this stage to ensure that there were no or very few cell doublets or aggregates and ensured that we only used samples with a minimum amount of debris to prevent problems with microfluidic droplet generation.

### In situ hybridization, microscopy, image processing and analysis

Male and female FVB/N mice aged 3–6 weeks were used for tissue isolation. Trigeminal and DRG were harvested and fresh frozen in OCT (Tissue-Tek). 10 μm sections were used for in situ hybridization. Multi-color in situ hybridization were performed using RNAscope Multiplex Fluorescent Assay (Advanced Cell Diagnostics) as instructed by the manufacturer. Single color in situ hybridization was performed as described previously [19]. Trpv1, Trpm8, and Tubb3 were detected using probes that recognize their respective 3’ UTR; Piezo2 was a mixture of three probes spanning the entire length of the transcript. Th was detected using a full length probe while Mrgprb4 was detected using a ~ 1kb coding sequence fragment.

Brightfield photomicrographs were obtained using an Aperio Scanscope CS system (Leica); images were compiled (cropped) in Photoshop CC (Adobe) and minor adjustments to brightness and contrast were applied to match background levels. Confocal microscopy (1 μm optical sections) was performed with a Nikon C2 Eclipse Ti (Nikon). All confocal images shown are collapsed (maximum projection) stacks of 10 individual optical sections. Images were cropped processed in Photoshop CC (Adobe) and minor adjustment of brightness and contrast were made for each label. Multiple sections (at least 4) from multi-color in situ hybridization were used to quantify the number of cells expressing a particular gene.

### Dropseq capture and sequencing of single-cell transcriptomes attached to microparticles

Single cells and barcoded beads (ChemGenes, Lot#011416B) were captured using a hydrophobically-treated microfluidic device (Nanoshift) as described by Macosko et al. [10].

Cell and bead concentrations were adjusted to between 100–160 cells / μl and 120–210 beads / μl. We note that from experiment to experiment droplet size varied; however, in general droplets were smaller than originally reported (generally around 0.5 nl), reducing the probability of multicell capture. Reverse transcription and cDNA libraries amplification (15 cycles) were as performed as described previously [10]. Next generation sequencing was performed using an Illumina Mi-Seq sequencer; sequencing reads were mapped to the mouse transcriptome using STAR and individual bar-coded libraries of transcripts were generated; 57.4% of sequences (range 48–62%) aligned to a single site in the mouse genome. The predicted number of single-cell transcriptomes attached to microparticles (STAMPs) was always considerably greater than the number that produced single-cell libraries, suggesting that only a fraction of the cell-preparation contains intact mRNA that efficiently hybridized with primer beads for cDNA synthesis. When samples containing 1000 predicted STAMPs were sequenced on a single MiSeq run only about 250 cells (range 227–309) were recovered with more than 1000 transcripts per cell. Increasing the number of predicted STAMPs per run to 2,600 only increased the number of libraries recovered with this level of coverage slightly to 365 ± 23 (s. e. m.) indicating that depth of coverage by sequencing begins to limited the quality (number of transcripts) of individual libraries when more than 1000 STAMPs are sequenced using MiSeq. Single cell libraries containing more than 1,000 unique transcripts were selected for analysis. Since the Dropseq primers include a UMI, this sequencing information is not distorted by differential amplification and is therefore directly related to expression level in an individual cell.

### Analysis of single cell sequence data

All analysis reported in this paper made use of the Seurat package developed by the Satija lab [13, 20]. In essence, we used the methods that they recommend in their tutorial for analyzing a dataset of 2,700 peripheral blood mononuclear cells for identification and display of clustering. Briefly, data were log normalized and center scaled; variable markers were identified and used for linear dimensional reduction (principle component analysis, PCA). Informative principle components were identified by plotting their standard deviations using the PCElbowPlot function of the Seurat package as described in the Satija lab tutorial. These principle components were used for clustering of STAMPS using a smart local moving algorithm [21]; multidimensional data were displayed using a tSNE 2-dimensional representation. A first round of clustering was performed using all the STAMPs that contained at least 1,000 transcripts; libraries containing less than 200 or more than 8,500 different genes and STAMPs containing more than 0.3% mitochondrial transcripts were excluded from analysis leaving 6998 single cell libraries. Clusters of STAMPs from somatosensory neurons were identified by their expression of known marker genes including Scn9a, Tubb3 and Snap25 as well as more specific transcripts like Trpv1, Trpm8 and Piezo2 and lack of expression of markers for other cells including Plp1, Mbp and Epcam. Somatosensory neurons were re-clustered using more stringent criteria for inclusion: 500–7,500 genes, 0.2% mitochondrial transcripts; genes expressed in less than 6 neurons were also excluded leaving a dataset of 3580 neurons and more than 15,000 genes.

We examined the stability of the clustering reported here using a variety of conditions and by using different clustering methods including tSNE based clustering [10]. Random selection of STAMPs demonstrated that the number of clusters (and their markers) was not changed once 1,500 to 2,000 neurons were included in the analysis thus increasing sample size incrementally beyond 3,500 neurons would be unlikely to change our conclusions. Similarly, clusters were not changed when different criteria were used for selection of variable genes or when the number of principle components used for analysis was varied between 15 and 25. tSNE based clustering [10] also yielded very similar results. More stringent selection of cells by requiring 900–7,500 different genes to be present in a STAMP, selectively reduced the number of S100b expressing neurons and resulted in collapse of this group of three clusters into a single cluster. In contrast, eliminating genes expressed in limited numbers of cells had little effect on clustering. For example, C11 and C12 each consist of less than 100 STAMPs; nonetheless these clusters were still separated when all genes present in less than 120 cells were excluded from the analysis. Indeed, very similar clustering was still observed even when genes expressed in less than 500 cells were eliminated (e.g. including genes like Trpm8) with concomitant reduction of the number of genes used from more than 15,000 to less than 6,000. In this analysis, itch clusters C11 and C12 merged, C7 cells were incorporated in other clusters and the Ntrk2 rich cluster C5 merged with C4. Thus the clusters that we identified appear extremely stable and are not simply determined by expression of marker genes that are expressed in that class of cells or by the clustering parameters chosen and methods that were used.

## Results and discussion

### Generation and analysis of sc-transcriptome data from trigeminal neurons

**Fig 1:**
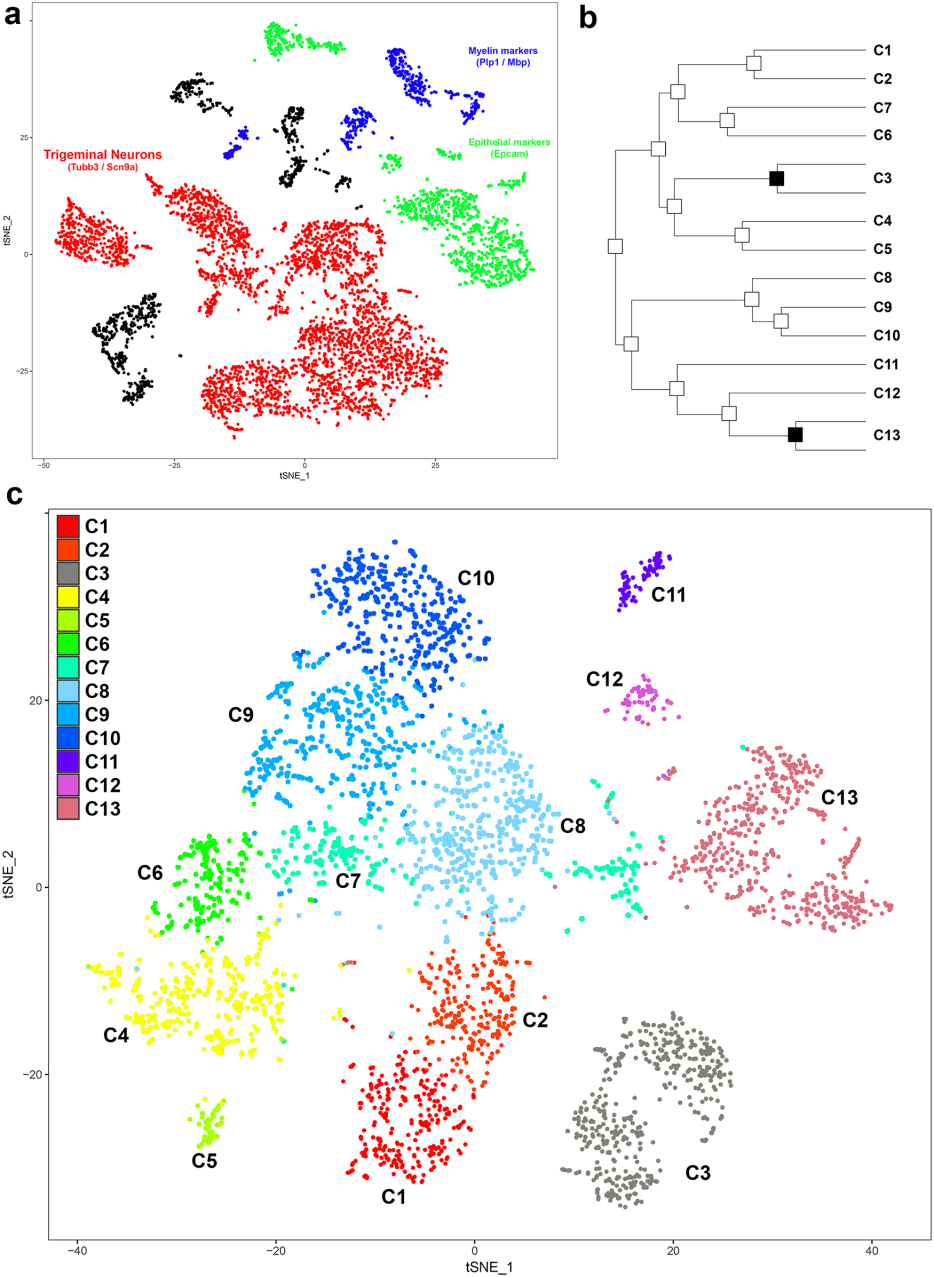
Unbiased clustering identifies 13 classes of trigeminal sensory neurons. (a) tSNE representation of 6998 STAMPS from Dropseq analysis of dissociated trigeminal ganglia separates clusters of sensory neurons (red) enriched in markers like Tubb3 and Scn9a from other associated cells including Epcam-positive epithelial cells (green) and cells producing components of myelin (e.g., Plp1 and Mbp, blue). (b) Trigeminal neuron STAMPs were re-clustered using a smart local moving algorithm to identify 15 potential classes of neurons. Nodes marked in black separated groups of neurons that did not express distinguishing markers; thus this analysis identified 13 clusters, which we have numbered according to their expression profiles and position in (c) the tSNE representation, where cells are color coded according to their cluster number.

**Fig 2:**
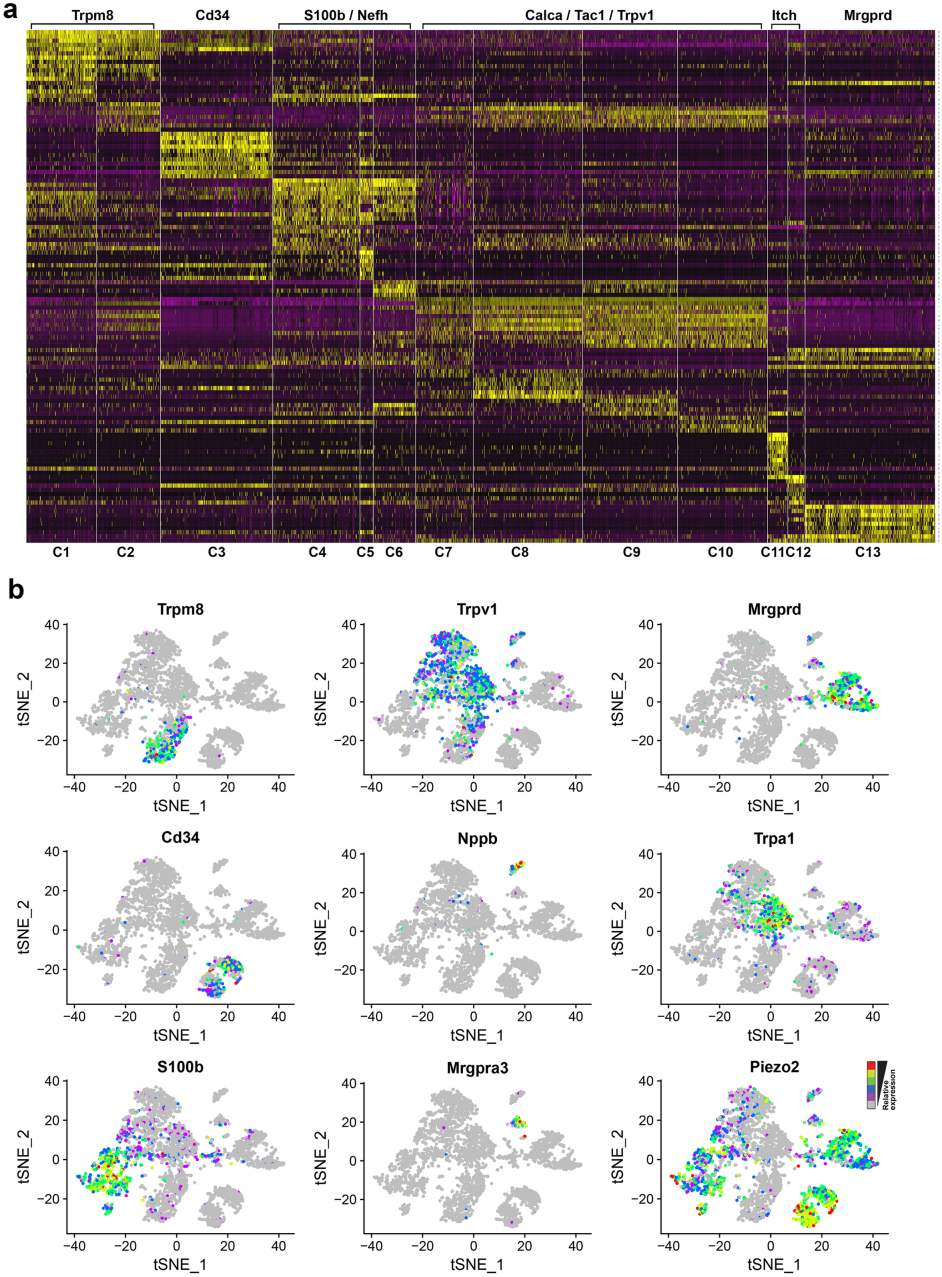
Classes of trigeminal neurons are distinguished by differential expression of many genes. (a) Heatmap showing the relative expression of a variety of markers in the trigeminal neuron STAMPS that have been ordered by cluster (numbered underneath the heatmap). Yellow represents high expression and purple low expression; the broad categories of neurons based on markers or function and referred to in the text are indicated above the heatmap. The list of genes used for this analysis is provided in S2 Table. (b) The expression profiles of select genes highlighted in this study are indicated in the tSNE-distribution of trigeminal neuron STAMPs with relative expression level color coded from grey to red. This representation emphasizes that genes like Nppb, Mrgpra3 and Mrgprd serve as markers of individual classes of neuron, whereas genes like Trpv1, Piezo2 and S100b are expressed in several different neural clusters.

We developed an efficient approach for bulk isolation of monodisperse neurons from the trigeminal ganglion and used the Dropseq technique [10] to obtain single cell sequencing of about 7,000 cells (referred to as STAMPs: single-cell transcriptomes attached to microparticles, see Methods for details). All data processing used the Seurat R-package [13, 20]. Unbiased cluster analysis (Fig 1a) using a smart local moving algorithm [21] grouped the sc-transcriptomes into more than 20 clusters that are mainly resolved in a 2-dimensional tSNE representation. STAMPs in several of the clusters expressed markers for non-neuronal cells and were well separated from the groups composed of sensory neurons. These non-somatosensory cells were removed from our dataset to leave about 3,500 trigeminal neurons that met a variety of criteria (see Methods for details). “Purified” trigeminal neurons were re-clustered to identify more than a dozen classes of somatosensory receptors (Fig 1b and 1c). It should be noted that just as described by the Satija group [13, 20], no perfect determination of stringency for cluster identification is possible and thus some of the identified nodes in the hierarchical clustering (Fig 1b) separate highly related groups of cells. Indeed comparison of expression of the top markers for each cluster (Fig 2a) and a more comprehensive examination of their gene expression profiles strongly suggest that the two nodes marked in Fig 1b do not distinguish separate classes of neurons and thus have been removed for subsequent discussion and analysis. All other neuronal clusters are supported by distinct expression profiles and likely reflect cellular diversity within the ganglion; we have numbered these thirteen clusters (Fig 1b and 1c) on the basis of their relationship in the tSNE plot and the markers they express (S1 Table, which contains statistical analysis for the 100 top markers of each cluster).

The trigeminal neuron-clusters that we identified could be tentatively assigned function based on the expression of genes that have been well studied previously [22]. These markers include ion channels involved in thermosensation (Trpm8 and Trpv1), mechanosensation (Piezo2) and chemical nociception (Trpa1), neuropeptides (Calca, Tac1, Nmb, Gal, Adcyap1 and Nppb) with roles in inflammation, pain and itch and G protein coupled receptors (GPCRs) e.g. P2ry1, Mrgprd and Mrgpra3, as well as structural markers and neurofilament associated genes related to neural size like Nefh, Nefm and Nefl (see S1 Fig).

Clusters C1 and C2 likely respond to cold as they express the cooling-activated ion-channel Trpm8 [5]. These Trpm8-clusters are distinguished from each other by higher expression of the neurofilament heavy chain, Nefh, in C1 and some neuropeptides (e.g. higher expression of galanin, Gal) in C2 suggesting that they likely have somewhat different roles in sensory detection. Cells in C3 express high levels of Piezo2 but low levels of Nefh. These neurons appear to represent affective touch C-fibers [23] based on expression of other markers including the GPCR, P2ry1 and the cell surface antigen Cd34 [17]. C4, C5 and C6 are Nefh-high neurons that all express the mechanosensory channel Piezo2 and thus are probably large myelinated neurons that contribute to different aspects of discriminative touch [23]. C7 is quite diverse and appears to represent a group of neurons that share markers both of nociceptors (e.g. Trpv1, Tac1 and Calca) as well as markers of large neurons including Thy1, the calcium binding protein S100b and Nefh. C8, C9 and C10 all express the heat and capsaicin sensitive ion channel Trpv1 as well as high levels of the neuropeptides Tac1 and Calca. These neuron classes likely have roles in heat sensation and nociception [24]. C11 is marked by a neuropeptide, Nppb, and other molecules with validated roles in itch [25], while C12 expresses Mrgpra3, a mas-related GPCR that also triggers itch [26]. C13 expresses another mas-related GPCR, Mrgprd and corresponds to a population of neurons that have been reported to play a role in noxious mechanosensation [27]. Thus our analysis of trigeminal Dropseq data segregates previously identified classes of somatosensory neurons with distinct functional roles and also provides a basis for exploring further specialization by identifying subdivisions between groups of neurons expressing classical markers.

One intriguing feature of this analysis is that there are relatively few genes that serve as true markers (i.e., genes only expressed in a single cluster, see Fig 2b). For the most part, genes that are highly enriched in one cluster tend to be also present in other classes of neurons or at least in scattered cells within other clusters. Importantly, STAMPs expressing the best markers including Nppb, Mrgprd, Mrgpra3 and Trpm8 are almost entirely contained within the gene-specific cluster or clusters, demonstrating that contamination of the Dropseq data e.g., by cell doublets or aggregates is not a significant problem complicating interpretation of the data. Thus we predict that scattered cells expressing cluster enriched genes, for the most part, reflect real variability in expression patterns across the different classes of trigeminal neurons.

### Translation of the trigeminal sc-transcriptome into cellular expression and identity

**Fig 3:**
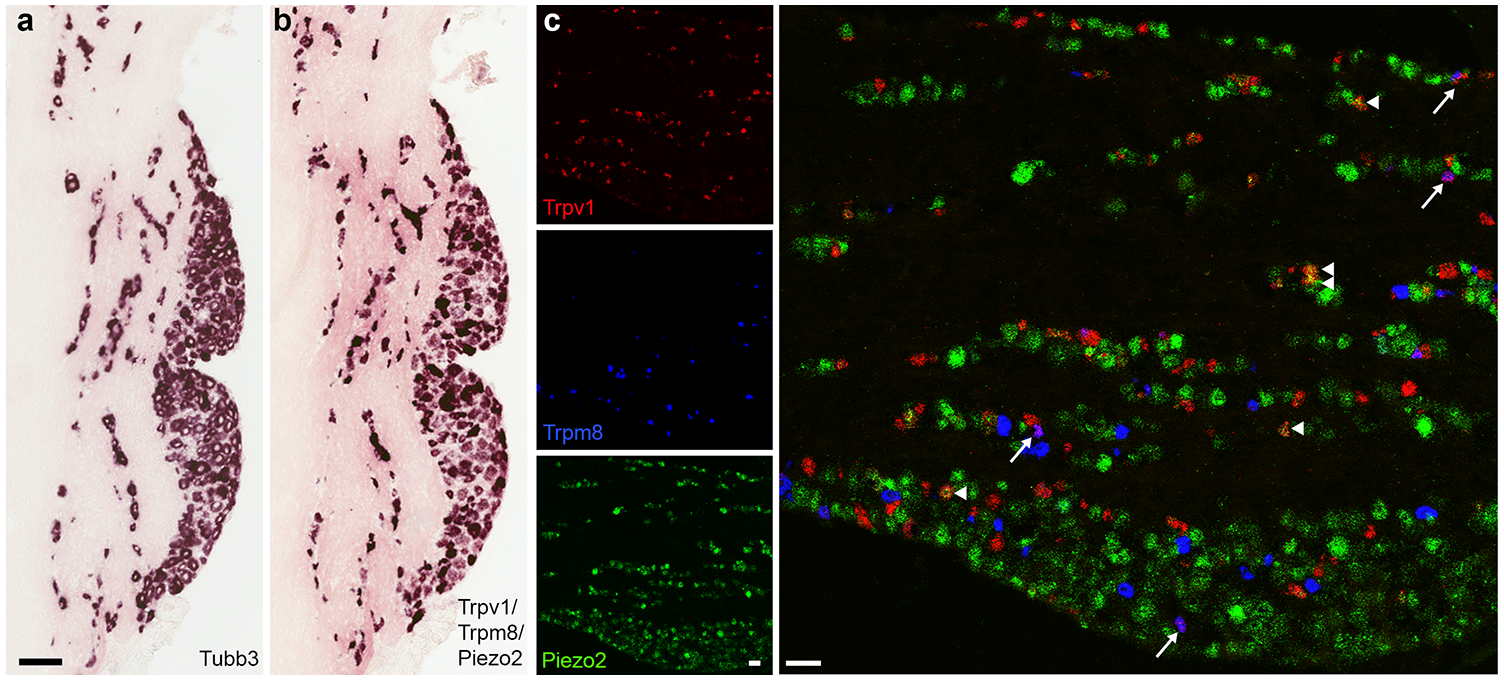
In situ hybridization localization of Trpv1, Trpm8 and Piezo2 in trigeminal neurons validates important predictions from sc-transcriptome analysis. Comparison of adjacent sections through the trigeminal ganglion (a, b) using single label ISH reveals (b) that a mixed probe for Trpv1, Trpm8 and Piezo2 labels essentially all the neurons detected in (a) using a probe to the highly expressed pan-neuronal marker Tubb3. (c) Three color, triple-label ISH demonstrates that Trpv1, Trpm8 and Piezo2 each mark large populations of neurons that do not express the other two transcripts, as predicted by Dropseq analysis (Fig 2b and S1 Fig). In keeping with the single cell data we also observed a subset of neurons that express both Trpv1 and Piezo2 (examples indicated by arrowheads) as well as neurons expressing both Trpv1 and Trpm8 (arrows); 165 of 718 Trpm8 and 1431 Trpv1 neurons across 29 sections were double positive. Scale-bars (a) 100 μm; (c) 50 μm.

The three ion-channels Trpv1, Trpm8 and Piezo2, which have key roles in thermosensation [3–7], mechanosensation [28, 29] and pain [24], each identifies a selective subset of neurons and neural classes. Interestingly, at least one of these transcripts is significantly expressed in every cluster (Fig 2b and S1 Fig). The expression level and number of STAMPs containing Trpv1 and Piezo2 exhibit variation between the different clusters (S1 Fig). For example, Piezo2 is highly expressed and present in most of the cells that make up C3 but is found at a lower level and in fewer cells in C11 and C12. Because Dropseq captures only one to a few thousand transcripts per cell, even moderately expressed genes (e.g., those expressed at 0.02–0.05% of total cellular transcripts) will only be detected in a fraction of the cells that express them. This is reflected in our data: whereas very highly expressed transcripts like β-actin, Actb or β3-tubulin, Tubb3 are reliably found in the vast majority of STAMPs (S2 Fig), genes such as synaptosomal-associated protein 25, Snap25 or the sensory neuron sodium channel, Scn9a [30], also expected to be found in most if not all trigeminal neurons, are present in a smaller subset (~ 80%) of the sc-libraries (S2 Fig). Therefore, based on their representation across clusters, we reasoned that at least one of Trpv1, Trpm8 and Piezo2 is likely to be expressed in any somatosensory neuron. Indeed, ISH using a mixed probe for these ion-channels closely resembles the neural complement detected by pan-neuronal Tubb3 in sections through the trigeminal ganglion (Fig 3a). Importantly, three color multi-label ISH (Fig 3b) confirms several other predictions from Dropseq, namely that most neurons uniquely express just one of these ion-channels but that there are a considerable number of neurons that co-express Piezo2 and Trpv1 and a smaller complement that contains both Trpm8 and Trpv1. In combination, these data support the differences in expression levels that we observed in the Dropseq analysis, substantiate Trpv1, Trpm8 and Piezo2 as widely expressed in the neural clusters where they are represented in a significant number of STAMPs and demonstrate that almost every trigeminal somatosensory neuron expresses at least one of these three genes.

The extent of Trpv1 and Trpm8 co-expression in trigeminal somatosensory neurons was somewhat unexpected given that these genes are generally considered markers for neurons specialized to respond to heating or cooling respectively [4, 6]. However, many cultured somatosensory neurons respond both to Trpv1 and Trpm8 agonists [7, 31] and a smaller number of DRG neurons have been shown to co-express both these classes of ion-channel [32]. Our data demonstrate that approx. 10% of Trpv1-neurons express the cooling sensitive ion channel Trpm8 and approx. 20% of Trpm8 neurons contain the heat and capsaicin activated ion channel Trpv1 (Fig 3c). Therefore we anticipate that thermosensation by trigeminal neurons may be more complex than the simple labeled line model proposed from behavioral studies of mice where Trpv1 or Trpm8 expressing neurons were selectively ablated [4, 6].

Intriguingly, the vast majority of the Trpv1 / Trpm8 double positive neurons fall within the Trpm8 clusters (C1 and C2, see Fig 2b and S3 Fig). However, the distribution of Trpv1 positive STAMPs within these clusters is relatively uniform in the tSNE plot (Fig 2b) indicating that these neurons do not constitute a distinct subset of Trpm8-positive neurons despite their expression of an additional thermosensitive channel. This implies that Trpv1 has the potential to modulate the function of a subset of Trpm8 neurons rather than acting as a marker for a subclass of Trpm8 neurons with a distinct and uniform gene expression profile. There are a few Trpm8-containing STAMPs that segregate into Trpv1 clusters (C8 and C9; S3 Fig). These neurons tend to express high levels of Calca, a marker of Trpv1 clusters, and low levels of Lxn, which is very prominent in Trpm8 expressing clusters (S3 Fig). Therefore a small minority of Trpv1, Trpm8 double positive trigeminal neurons may be heat or pain sensitive cells (Trpv1-nociceptors) that express the cool responsive channel Trpm8.

**Fig 4:**
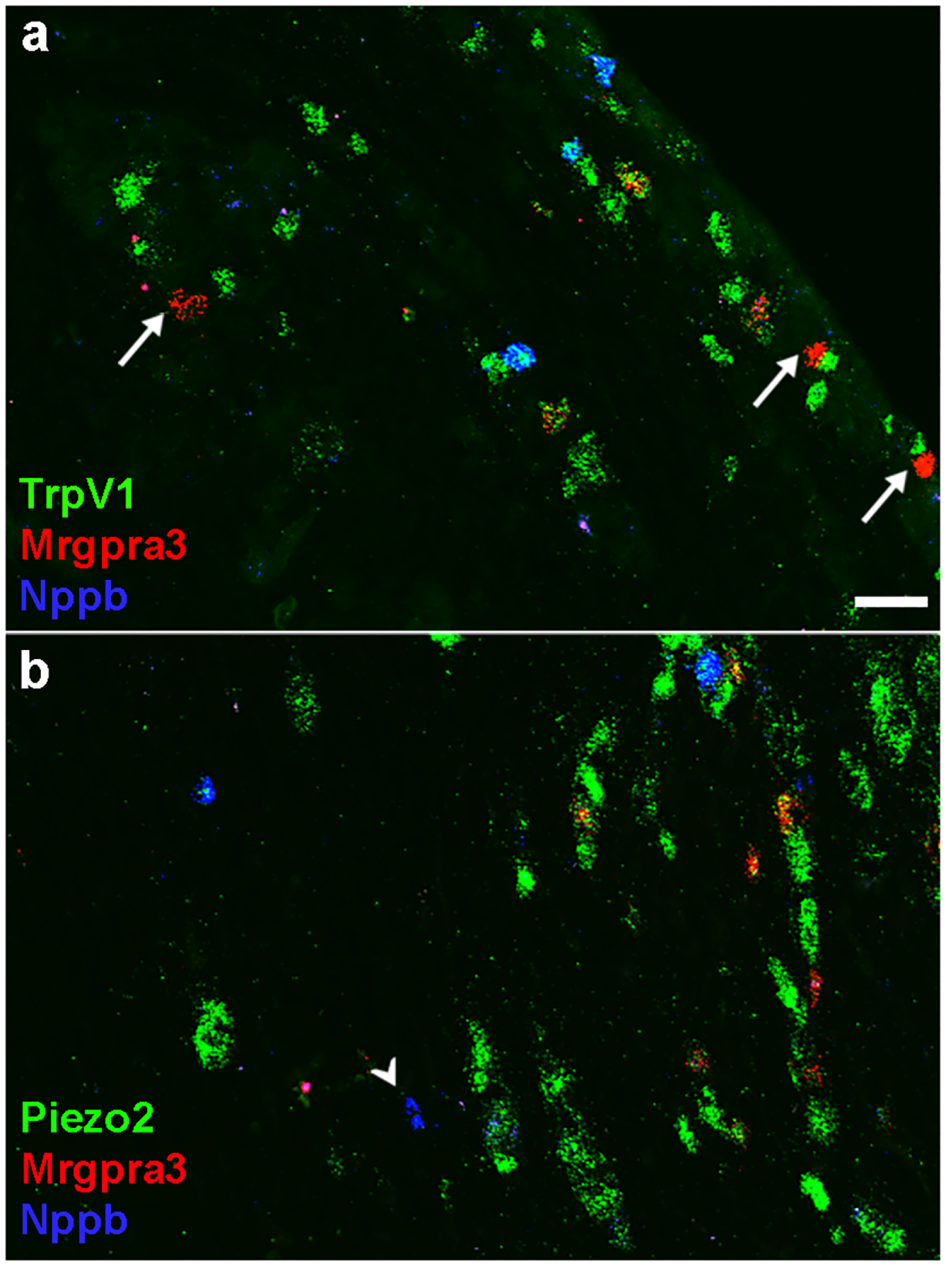
ISH localization of Nppb, Mrgpra3, Trpv1 and Piezo2 highlight the difficulty of assessing penetrance of low to moderate level gene expression in sc-transcriptome data. Representative sections through trigeminal ganglia subjected to three color, triple label ISH for (a) Nppb, Mrgpra3 and Trpv1 illustrate that all neurons expressing Nppb (blue) are also positive for Trpv1 (green); in contrast only a subset of Mrgpra3 neurons (red) co-express Trpv1; arrows indicate Mrgpra3 neurons not expressing Trpv1. (b) Trigeminal ganglion neurons expressing Mrgpra3 (red) all co-express Piezo2 (green); most Nppb (blue) also co-express Piezo2; arrowhead highlights a single Nppb positive, Piezo2 negative neuron. Note that Nppb and Mrgpra3 label almost completely non-overlapping subsets of neurons; scale bar 50 μm.

Trpv1, Trpm8 and Piezo2 expression patterns (Fig 3) not only reveal important features of trigeminal neural diversity but highlight two important considerations that apply to single cell sequencing. The first concerns under-sampling of sc-transcriptome and the consequent sparseness of the data. Notably, the ISH data demonstrate that these ion-channels essentially tile the full repertoire of trigeminal neurons, yet only ~75% of STAMPs contain at least one copy of any of these three genes and in some clusters only a small proportion of STAMPs express a given gene (S1 Fig). Therefore presence of a transcript in a subset of a class of neurons is ambiguous: the gene may really be expressed in a subset (as is the case for Trpv1 in Trpm8 neurons, Fig 3) or may be broadly expressed at a low level. This is well illustrated in our data where, Trpv1 and Piezo2 are present in subsets of STAMPs from both clusters C11 and C12 (Fig 2). We used in situ hybridization to examine co-localization of Trpv1 or Piezo2 with Nppb (a marker for C11) and Mrgrpra3 (C12). The ISH data demonstrate that only a fraction of the Mrgpra3 neurons but all Nppb cells express Trpv1 (Fig 4a). In contrast, all Mrgpra3 and most Nppb cells express the mechanosensitive ion channel Piezo2 (Fig 4b). Thus multi-labeling techniques can rapidly resolve ambiguities and substantially enhance interpretation of unbiased cluster analysis.

The second issue relates to the assumption that approaches like Dropseq provide a quantitative analysis of a cell population. In many cases this is likely to be true; however, trigeminal neurons are heterogeneous with considerable morphological variation including soma size [18]. Therefore techniques used in the isolation of single neurons and differences in efficiency of mRNA capture may affect representation of different classes of trigeminal neurons. Indeed the triple label ISH identifying Trpv1, Trpm8 and Piezo2 positive neurons exposed a marked difference between the numbers of positive cells that would be predicted from Dropseq data and the observed frequency of positive cells (Figs 2b and 3). ISH results demonstrate that about 2.5-times as many cells express Piezo2 as Trpv1 (Fig 3c). By contrast, the number of Piezo2 and Trpv1 positive sc-transcriptomes as well as the number of cells in clusters that prominently express these gene predict that relatively similar numbers of neurons should express these transcripts (Fig 3a and S3 Table). Thus the trigeminal sc-transcriptome does not quantitatively reflect the trigeminal ganglion.

**Fig 5:**
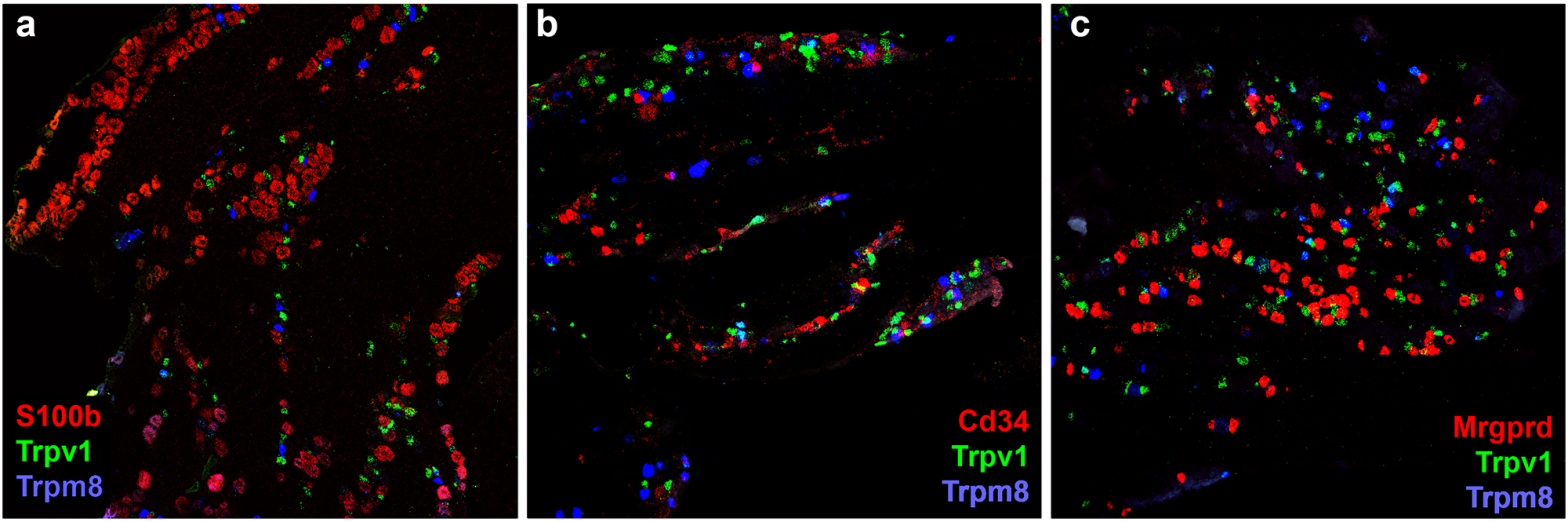
ISH localization markers reveals bias in representation of some cell classes in the Dropseq data. Triple label ISH of sections from the trigeminal using probes for Trpv1 (green) and Trpm8 (blue) together with (a) S100b, (b) Cd34 and (c) Mrgprd (red) support extensive segregation of these three markers from Trpv1 and Trpm8 in trigeminal neurons in keeping with their expression profiles in non-overlapping clusters of neurons. However, the ISH data illustrate discrepancies between the relative numbers of hybridizing cells with probes to S100b and Mrgprd and the representation of STAMPs expressing these markers in Dropseq data. Cell counts are quantitated in S3 Table and demonstrate that STAMPs expressing Mrgprd and S100b are underrepresented in the Dropseq data.

To better understand the nature and extent of this bias, we carried out additional multilabel in situ hybridizations using markers of several additional clusters and groups of clusters including S100b, Cd34 and Mrgprd (Fig 5). Quantitation of ISH data (S3 Table) demonstrate that large cells, represented by expression of S100b (and Piezo2) as well as smaller cells expressing Mrgprd (and Piezo2) are underrepresented in the Dropseq analysis relative to ISH. One possibility is that STAMPs from these cells are not as rich in transcripts and genes as those of other cells and are thus filtered from our analysis. Indeed, when we adjusted the stringency for sc-transcriptome inclusion by requiring that a library contains at least 900 genes, the number of S100b neurons in the clustering falls relative to other clusters. However, we also suspect that the single cell preparation and, perhaps instability of flowing the large S100b positive cells through the narrow channels of the Dropseq device have a role in the underrepresentation of particular cells. Although there is no reason to believe that important subtypes of trigeminal neurons are completely absent from our analysis, the significant loss of the large diameter mechanosensors reduces the power of sc-transcriptome analysis for identifying subclasses of these neurons and we predict that increasing the number of these cells would likely resolve more than 3 classes of large diameter mechanosensors (see [33]).

### Molecular classes of trigeminal neurons and their relationship to somatosensory neuron classes identified in the DRG

Five of the thirteen types of neurons identified by clustering trigeminal sc-transcriptome data corresponded closely to classes previously described amongst DRG neurons [15–17]. Interestingly, these are the most distinct clusters and are well separated in the tSNE representation (Figs 1b and 2). They include C13 (marked by Mrgprd, Fig 5c), C11, cells likely involved in mediating inflammatory itch (Fig 4) and C5, a distinct group of Ntrk2, Nefh, S100b positive neurons that closely corresponds to the large, mechanosensitive Ntrk2 neurons that were identified in the DRG [16, 17]. Two other classes of neurons, C3 (marked by Cd34, Fig 5b) and C12 (expressing Mrgpra3, Fig 4) also very selectively express some of the markers of neuronal types that were identified in the DRG [16, 17]. However, sc-transcriptome data indicate that there are substantial differences in the expression pattern of diagnostic genes in these neurons between the DRG and trigeminal ganglia.

**Fig 6:**
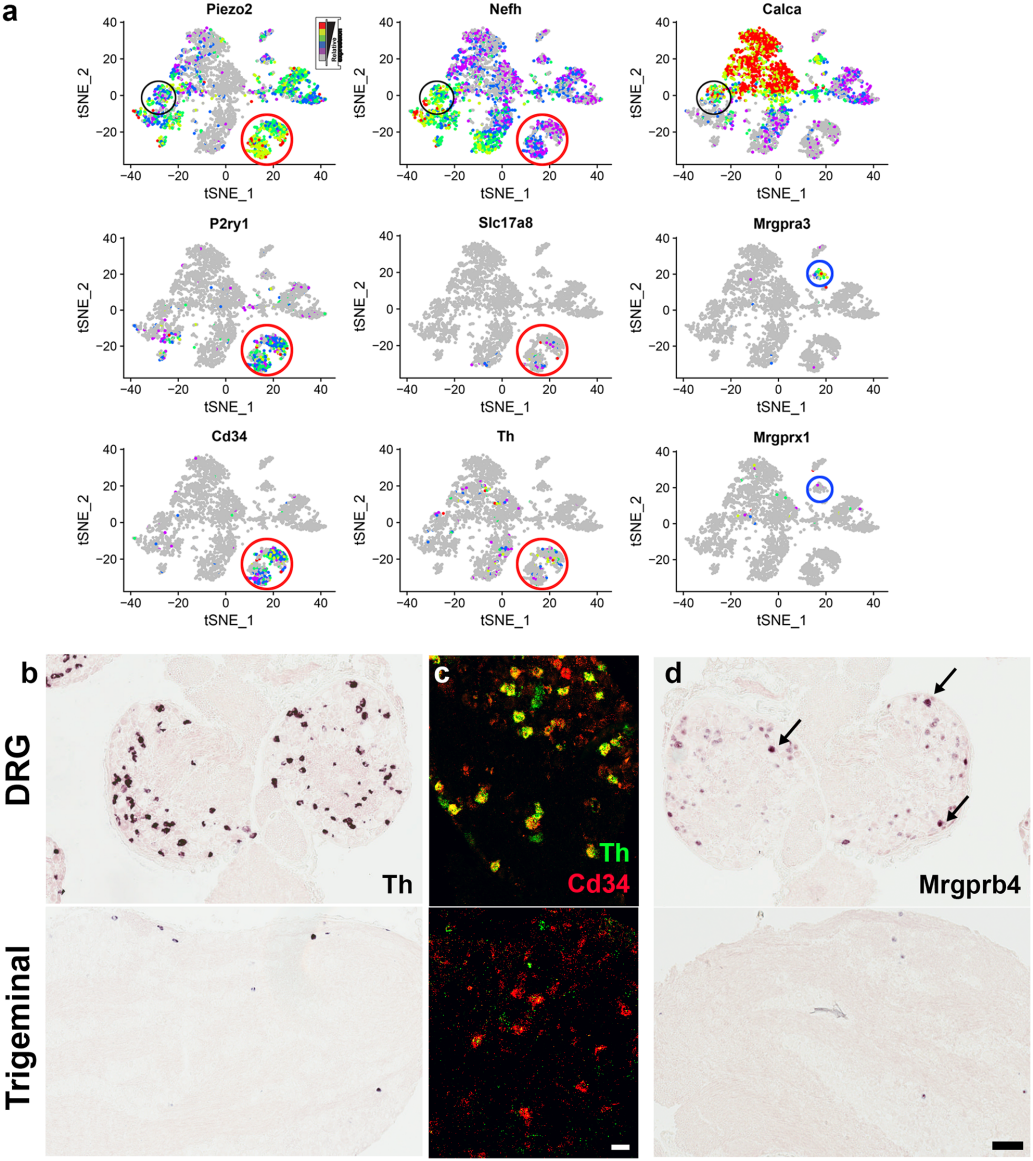
Expression of marker genes that differ between DRG and trigeminal ganglia. (a) Expression profiles of genes in trigeminal Dropseq data are shown in tSNE plots. In the trigeminal ganglion, putative affective touch neurons (red circles) expressing high levels of the mechanosensory ion-channel Piezo2 but low levels of Nefh are marked by Cd34 and P2ry1 but not by Slc17a8 orTh. Similarly the Mrgpra3 cluster (C12, blue circles) is not enriched in Mrgprx1 expression. Black circles highlight a class of large diameter (Nefh, high), mechanosensory (Piezo2, high) neurons that co-express Calca. (b-d) Representative sections of DRG (upper panels) and the trigeminal ganglion were subjected to ISH. (b) Single label ISH confirms that Th is prominently expressed in many DRG neurons but is only found in a much smaller subset of trigeminal cells. (c) Double label ISH reveals extensive co-labeling of DRG neurons expressing Cd34 (red) with Th (green) but demonstrates that the majority of trigeminal Cd34 neurons do not contain detectable Th. (d) ISH demonstrates that Mrgprb4 is prominently expressed in a small subset of DRG neurons (arrows), with weaker expression in many additional cells (upper panel). By contrast, Mrgprb4 is only weakly expressed in a small subset of trigeminal neurons. Scale bars; (c) 50 μm; (b, d) 100 μm.

Cluster C3 is a group of neurons that express high levels of the mechanosensitive channel Piezo2 but low levels of Nefh (Fig 6a). They are the main group of cells that express the metabotropic purinergic receptor P2ry1 (Fig 6a) and contain other transcripts that in the DRG are associated with a class of neurons marked by expression of tyrosine hydroxylase (Th) and the vesicular glutamate transporter VGlut3 (Slc17a8). The shared markers include the genes Cd34 and Gm7271([16, 17] and S1 Table). Notably neither Th nor Slc17a8 is highly represented or particularly selective for cluster C3 in the trigeminal Dropseq data (Fig 6a and S1 Table). We reasoned that this could be because there are differences in gene expression in the DRG and trigeminal ganglia, or alternatively that it reflects dropout of these transcripts from STAMPs and the lower depth of sequencing in our data. To distinguish these possibilities we used in situ hybridization to examine the expression of Th and Cd34 in both the trigeminal ganglion and DRG (Fig 6b and 6c). Our results confirm extensive co-expression of Th and Cd34 in the DRG, but also demonstrate that Th is expressed in a much smaller number of cells in the trigeminal ganglion, and show that in this ganglion, Cd34 positive neurons do not generally contain Th, exactly as predicted by the Dropseq data.

Cluster C12 is marked by expression of the G-protein coupled receptor Mrgpra3 (Fig 6a). In the DRG, it was reported that Mrgprx1 [16, 17] also marks this class of neurons, whereas trigeminal Dropseq data indicate that Mrgprx1 is predominantly found in other clusters (Fig 6a). Another Mas-related GPCR, Mrgprb4 was proposed to subdivide this class of neurons in the DRG [16] but is absent altogether from our trigeminal sequence data. We used in situ hybridization to examine the expression of Mrgprb4 in both ganglia (Fig 6d). Notably, whereas Mrgprb4 is expressed in a large subset of DRG ganglion neurons, it is only detected in a much smaller number of trigeminal neurons. Taken together, these data concentrating just on a few genes that have been singled out as important markers of classes of DRG neurons demonstrate that there are significant differences in gene expression between related neuron populations in the DRG and trigeminal ganglia.

Other neuronal classes identified from clustering trigeminal sc-transciptomes less obviously matched the groupings assigned for the DRG. In particular, Clusters C1 and C2, well resolved groups of neurons prominently expressing Trpm8 (Fig 2) were not distinguished by DRG single cell sequencing [15–17]. Moreover, although our analysis identified classes of trigeminal neurons that are rich in neuropeptides including clusters C7, C8, C9 and C10 (Fig 2a and S1 Fig) their expression profiles (Fig 2a and S1 Table) are not concordant with any class of neuron defined by DRG single cell analysis [15–17]. Similarly apart from the well resolved Ntrk2 class of Nefh/S100b cells (C5), other groups of Nefh/S100b neurons identified in the present study (C4 and C6) show significant differences from the various groupings that were resolved in the DRG (compare S1 Table and [15–17]).

**Fig 7:**
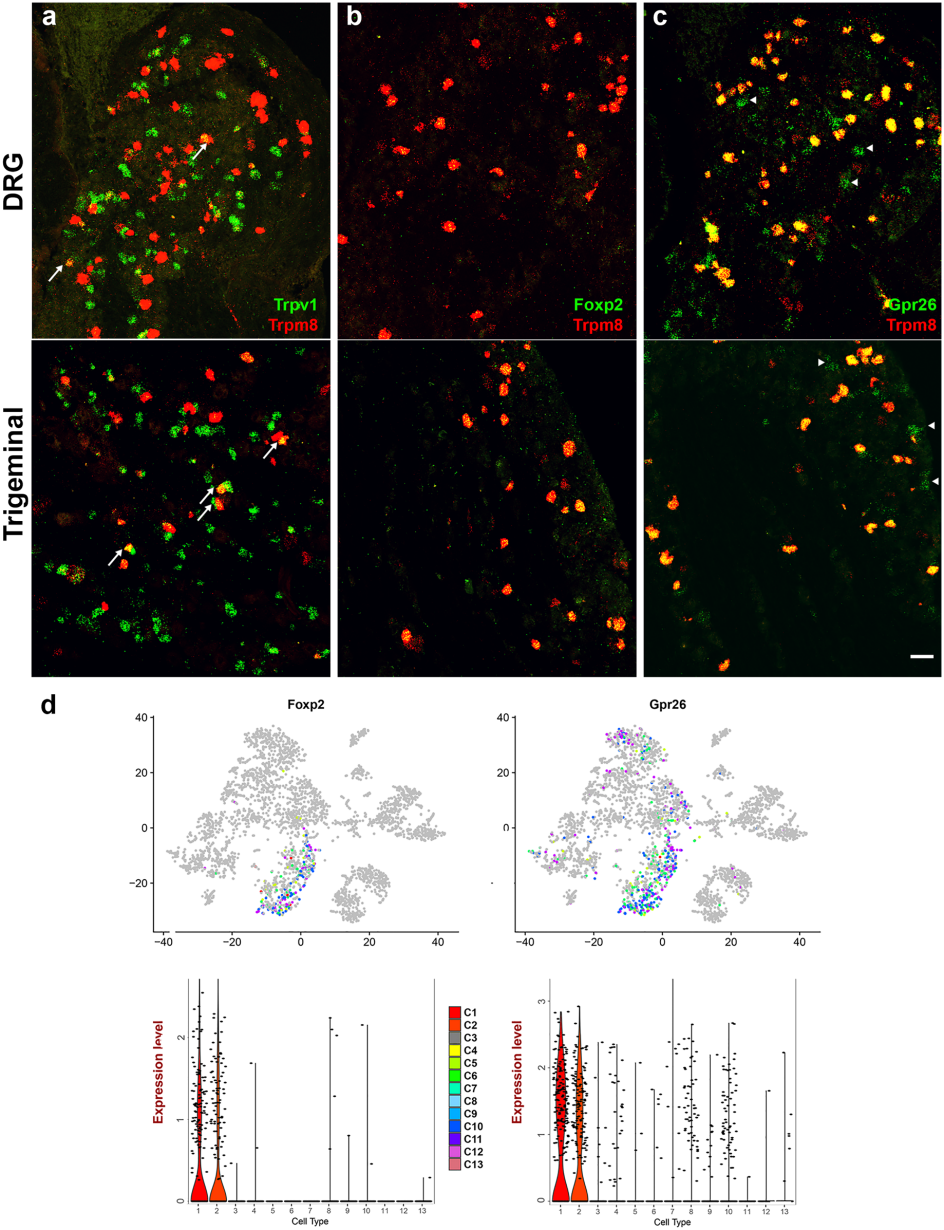
Trpm8 neurons have related expression profiles in the DRG and trigeminal ganglion. (a-c) Representative sections of DRG (upper panels) and trigeminal ganglion (lower panels) probed by double label ISH illustrate co-expression of Trpm8 (red) with (a) Trpv1, (b) Foxp2 and (c) Gpr26 (green). Note the similarity of expression patterns between the different somatosensory ganglia: (a) there is limited overlap between Trpv1 and Trpm8 in both ganglia (arrows) with 28 double positive in 534 Trpv1 and 265 Trpm8 cells across 7 sections in the DRG (5% of Trpv1 and 11% of Trpm8 neurons); for comparison a higher double positive ratio was observed in the trigeminal ganglion (12% of Trpv1 and 23% of Trpm8 cells, Fig 3; these differences are significant (p < 10^−4^, two tailed z-test). (b) Foxp2 is expressed with high specificity but at a low level in Trpm8 cells in both types of ganglion; (c) Gpr26 is another good marker of Trpm8 cells in both ganglia but is also expressed in cells that do not contain the cooling sensitive ion-channel (arrowheads); scale bar 50 μm. (d) Expression profiles of Foxp2 and Gpr26 in STAMPs indicated in tSNE (upper panels) and violin plots (lower panels) demonstrate that Dropseq analysis closely matches the ISH localization data.

**Fig 8:**
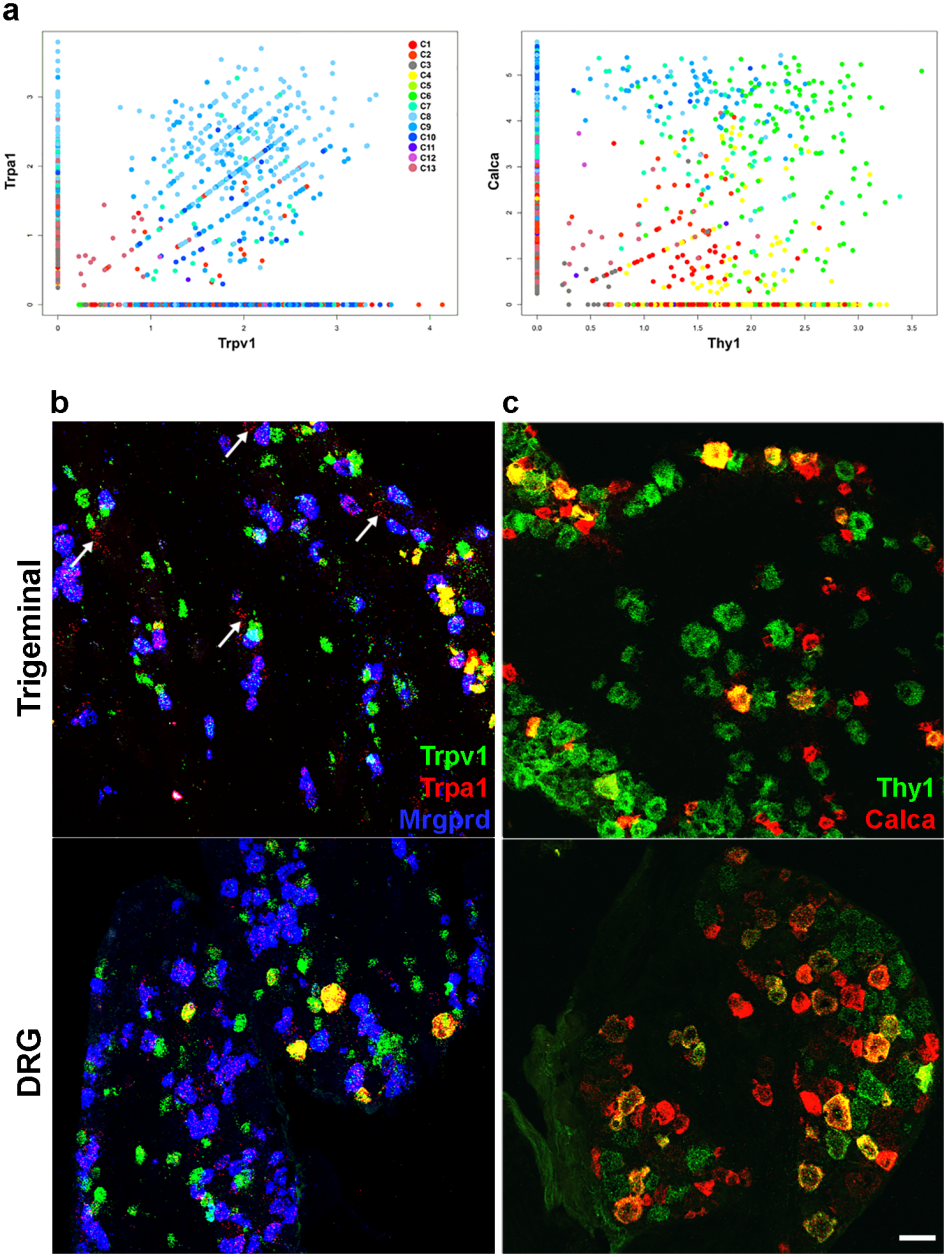
Co-expression patterns predicted by trigeminal Dropseq are found in DRG as well as trigeminal ganglia. (a) Relative expression level of genes in positive STAMPs plotted against each other and color coded according to cluster highlights co-expression of Trpv1 and Trpa1 in cluster C8 and co-expression of Thy1 and Calca in cluster C6 with far less co-expression outside these cells (see also Fig 2 and S4 Fig). (b,c) Representative sections of trigeminal (upper panels) and DRG (lower panels) probed by in situ hybridization illustrate (b) that a subset of Trpv1 neurons (green) express high levels of Trpa1 (red). However, many neurons, strongly positive for Trpv1 are Trpa1 negative in both types of ganglion. Note that a large subset of neurons expressing Mrgprd (blue) express a lower level of Trpa1 and that a smaller subset are also positive for Trpv1 in both trigeminal and DRG. A separate group of cells that do not express Trpv1 or Mrgprd are weakly positive for Trpa1 (arrows in the trigeminal ganglion) as predicted from the sc-transcriptome analysis (see also S1 Fig); these neurons may be less prominent in the DRG. (c) Thy1 (green) and Calca (red) are co-expressed primarily in large neurons in both the DRG and trigeminal ganglion; scale bar 50 μm.

Do these differences in neuronal classes reflect biology or the different approaches used? I.e., do the trigeminal and DRG contain some fundamentally different neuron types; or does variation between neurons within the divergent clusters mean that under-sampling of cells and/or transcripts prevents accurate classification of the full neural-repertoire? Clearly, it is likely that a combination of these explanations explains the differences. For example, the trigeminal ganglion does not contain proprioceptive neurons that were prominent in the DRG analysis [15–17]. Conversely, because Trpm8 cells have previously been implicated as selectively responsible for behavioral responses to cooling [4, 5], we suspected that under-sampling of Trpm8 neurons in the DRG likely prevented clustering of these neurons in the earlier analyses [15–17]. Indeed, in situ hybridization detects significant populations of Trpm8 neurons in both DRG and trigeminal ganglia (Fig 7) and demonstrates similar overlap with other genes that are present in C1 and C2 in the trigeminal dataset. These include Trpv1 (Fig 7a), the transcription factor Foxp2 (Fig 7b), which we discovered is expressed at a low level but very closely matches Trpm8 expression and a novel G protein coupled receptor, Gpr26, which we show to be present in most Trpm8 cells as well as additional cells outside the Trpm8-clusters (Fig 7c), as predicted from the Dropseq analysis (Fig 7d). Thus we conclude that Trpm8 neurons were missed from previous studies [15–17] because not enough of these cells were sequenced. Quantitation of ISH data shows that the proportion of Trpv1, Trpm8 double positive neurons is higher in trigeminal than in DRG (Fig 7a). Nonetheless, although there may be differences in numbers and specific gene expression profiles between the neurons in the trigeminal and the DRG, we anticipate that most if not all classes distinguished by sc-transcriptome analysis of the trigeminal ganglion are shared across these somatosensory ganglia. Indeed, additional ISH data (Fig 8) support this view. First, Dropseq analysis identified a subset of Trpv1-neurons that co-expresses Trpa1 in the trigeminal ganglion (Fig 2b and Fig 8a); ISH demonstrates that such neurons are found both in the trigeminal and DRG (Fig 8b). Interestingly, Dropseq analysis suggests (S1 Fig) that Trpa1 may be expressed at a low level in Mrgprd cells. Triple label ISH confirms this in both the trigeminal and DRG (Fig 8b), demonstrates that Trpa1 expression subdivides Mrgprd cells and shows that some of the Mrgprd / Trpa1 neurons also expressTrpv1. In addition, Trpa1 is expressed in some cells that contain neither Trpv1 nor Mrgprd (arrowed in Fig 8b); these cells appear more prominent in the trigeminal ganglion and are also detected in the Dropseq data (S1 Fig). Second, single cell data point to a class of large mechanosensory neurons that express Calca (Fig 6a). Double label ISH demonstrates that a subset of large neurons expressing the marker Thy1 are Calca positive in both sets of ganglia (Fig 8c). Since Thy1 and Calca expression primarily overlap in the neurons that make up cluster C6 (Fig 8a and S4 Fig) we surmise that this cluster is composed of large mechanosensory cells and is present in both the trigeminal and DRG.

### Concluding remarks

We carried out large-scale single cell sequencing of the trigeminal ganglion as a means to distinguish classes of neurons that might be involved in its specialized somatosensory functions [18]. As expected, our analysis identified several classes that were also found in the DRG including populations that mediate itch [15–17] and uncovered new classes of neurons in the trigeminal ganglion. For example, two clusters expressing Trpm8 that were not seen in previous datasets likely function in cold detection, and the broad group of nociceptors prominently expressing Trpv1, Calca and Tac1 is subdivided into 4 distinct clusters in our analysis and differ from the classes of peptidergic neurons identified in the DRG [15–17]. However, differences in approach appear to account for some of this variation (Figs 7 and 8) and perhaps surprisingly, the lumbar DRG, which primarily innervate skin and muscle, appear to largely share the neuronal classes found in the trigeminal ganglion with its much more varied sensory input e.g. oral mucosa, cornea, meninges, etc.

There remain some questions as to the exact significance of the different clusters that are identified in this type of analysis. For example, is each one a distinct class of neurons that together have a common functional role? In some cases, the clusters are very homogeneous and likely simply represent spatially separated parallel lines of sensory input. For example, the small, well separated cluster C11 selectively expresses a number of genes associated with inflammatory itch. However, a major surprise was that genes including Trpv1 [3, 4, 24] and Piezo2 [28, 29] are often broadly expressed across clusters that likely have quite different sensory roles. Double label ISH using Trpv1 (Figs 3 and 4), demonstrates that this gene, which is often referred to as a marker for heat sensitive neurons, generates potential for functional diversity within classes of neurons mediating responses to cold and pruritogens. Similar cluster specific heterogeneity was observed for the mechanosensitive ion channel Piezo2 (Fig 3) and other genes like Trpa1 (Fig 8) and the inflammation and pain related neuropeptide Calca (S1 and S3 Figs). Thus, our results demonstrate that many of the trigeminal neuronal clusters encompass groups of receptor cells with a range of potential sensitivities and tuning properties. It is tempting to speculate that this type of variation helps explain the four separate types of cold responsive neurons, including a class that only responds to cold after injury, described recently [8]. Finally, the variation in expression of genes that have been considered markers for select classes of somatosensory neurons adds important nuance to the concept of labeled lines as a primary mechanism of somatosensory signaling and confirms electrophysiological findings describing polymodal nociceptors [2].

The trigeminal sc-transcriptome dataset provides a valuable resource for performing in silico expression analysis. This can complement other approaches in assigning roles for genes and cells in somatosensation, potentially cementing earlier deductions as well as raising questions about the roles and interactions of specific genes. For example, the sodium channel, Scn10a (Nav1.8) has been widely used as a marker for a broad subset of c-fiber nociceptors [34, 35]. However, its distribution is quite variable across the different classes of trigeminal neurons (S4 Fig), with no expression in the clusters that likely respond to cooling and express Trpm8. One of the phenotypes of Scn10a knockout mice is a dramatically reduced response to painful cold but unchanged avoidance of cool [35]. Consequently, clusters C1 and C2 (and therefore also Trpm8) are not likely to be the principle detectors of painful cold. Another sodium channel, Scn1a, which has recently been implicated in painful mechanosensation [36], is expressed in classes of mechanosensory neurons but is also prominent in Trpm8 cells (S4 Fig), insinuating a role for this channel in cool temperature responses. Similarly, the membrane protein, Tmem100 was shown to be an important modulator of interactions between Trpv1 and Trpa1 [37]. Interestingly, in the trigeminal ganglion, the expression pattern of Tmem100 is not restricted to the neurons co-expressing these two Trp-channels (S4 Fig) again suggesting additional roles for Tmem100. The raw dataset and Seurat clustering associated with our analysis has been deposited in GEO (Accession number: GSE101984).

Taken together, our results expose considerable diversity amongst somatosensory neurons in part by defining new classes of nociceptors and identifying distinctions between DRG and trigeminal neurons, but most notably by demonstrating heterogeneity even amongst neurons that co-cluster based on their gene expression profile. This heterogeneity means that direct identification of single markers for dissecting expression-function relationships may not be possible for all classes of somatosensory neurons. However, our data suggest that combinatorial methods should allow targeting of most clusters and in the future this approach could also be used to investigate the role of subsets of cells like the Trpm8 neurons that co-express Trpv1. Finally, it is likely that new developments that increase read depth as well as approaches like nuclear-RNA sequencing [38], which may reduce biases in cell isolation, will further boost the power of sc-transcriptomics for studying sensory detection. We anticipate that in the near future such improvements may even allow monitoring of activity dependent changes in gene expression to be used as a means to directly probe the function of individual cells.

## Supporting information

S1 Fig Cluster specific expression profiles of select genes reported to play key roles in somatosensation. (a) Dot plots depicting transcript representation in STAMPs comprising the 13 clusters C1-13. The diameter of the dot is related to the fraction of STAMPs in that cluster expressing the gene; the shade of the dot reflects the relative level of expression of a transcript in a cluster. (b) Violin plot display of expression data for the same genes: gene expression for individual STAMPs is represented by dots; vertical lines show the maximum expression level in a cluster while colored curves depict the significant expression in individual clusters. The colors are identical to those used in Fig 1c. Note that all these genes exhibit marked differences in expression between the different classes of neurons identified in the clustering analysis.
(TIF)

S2 Fig Prominent pan-neuronal markers illustrate the problem of “dropout” of even highly expressed genes in individual STAMPs. Three different representations of gene expression demonstrate that very highly expressed genes such as Actb or Tubb3 are found in almost every STAMP used in our analysis (98% and 94% respectively). In contrast, other highly expressed genes Snap25 and Scn9a that are expected to be present in all somatosensory neurons are absent from a substantial number of STAMPS as indicated in (a) the tSNE representation, (b) the dot plot and (c) the violin plot; Snap25 and Scn9a are present in 81% and 79% of STAMPs. Note that the dropout of Snap25 and Scn9a is cluster dependent and the cluster specific patterns of expression for these two genes do not match as can be seen in (a) the tSNE representation. Dot plot analysis (b) shows that dropout is greater (smaller dots) for clusters where average relative expression is lower (paler shade). This is also apparent from the violin plot (c) where the extent of the null population is related to the size of the lower bulge. Note neither the number of genes (nGene) nor the number of UMIs (nUMI) is dramatically different between clusters.
(TIF)

S3 Fig Trpm8 neurons that express Trpv1 primarily localize to the Trpm8 clusters C1 and C2. Shown in each graph are the relative expression of a markers and Trpm8 within STAMPs that are positive for either gene; the points represent a single STAMP and are color coded according to cluster using the colors shown in Fig 1c. (a) STAMPs co-expressing Trpm8 and Trpv1 are mainly clustered in C1 (red) and C2 (orange) with only a few STAMPs from the main Trpv1 clusters (blue) being positive for both genes. Most Trpm8 neurons that do not cluster in C1 and C2 (circled, including those that express Trpv1) express high levels of (b) Calca, a marker of peptidergic nociceptors and low levels of (c) Lxn which is prominently expressed in almost all C1 and C2 neurons.
(TIF)

S4 Fig Predictive value of in silico analysis illustrated by expression profiles of functionally important genes. Expression profiles for marker gene Thy1, the functionally important sodium channels Scn10a and Scn1a as well as the transmembrane protein Tmem100 represented in t tSNE analyses (upper panels) and violin plots (lower panels). Thy1 is primarily expressed in neurons that express the highest levels of Nefh, Nefm and Nefl and thus large diameter neurons. Note Scn10a which is required for aversive responses to noxious cold is not expressed in the Trpm8 clusters C1 and C2 (starred). In contrast, Scn1a is prominently expressed in these neurons. Tmem100 was recently reported to mediate interactions of Trpv1 and Trpa1 that are important for pain sensation. However, whereas Trpv1 and Trpa1 co-expression is strongest in C8 (starred), Tmem100 is prominently expressed in other clusters including C6, which are a group of large (Thy1 high), Piezo2 positive neurons likely involved in mechanosensation that generally do not express either Trpv1 or Trpa1 but do express Calca (see Fig 8).
(TIF)

S1 Table Top markers for each cluster. The 100 top markers for each cluster compared to total expression in all other clusters were determined using a combined likelihood ratio test [39] within Seurat. This test provides statistical data (P values, p_val) related to the chance that a gene is enriched in a cluster as well as values for expression level difference (avg_diff) and positive fraction of cells inside (pct.1) and outside (pct.2) the cluster. Each cluster has been provided a separate tab to aid sorting.
(XLSX)

S2 Table List of genes used for heatmap shown in Fig 2. Genes are shown in the order displayed in the heatmap (Fig 2); the genes and order were chosen semi-empirically from top markers to highlight the similarities and differences between clusters.
(DOCX)

S3 Table ISH demonstrates biases in Dropseq representation of different neural classes. Counts of positive cells using markers for trigeminal clusters, pan-neuronal genes and groups of neurons from multi-label ISH were compared with counts of positive cells in the Dropseq dataset. (a) Quantitation of positive cells in sections of the trigeminal ganglion probed by ISH with the pan-neuronal marker Scn9a in combination with one or two other markers of trigeminal neurons (see Methods). Positive STAMPs and STAMPs in the major clusters positive for a marker are also shown. We calculated z-scores between normally distributed proportions to determine whether differences between ISH and Dropseq data are significant; P values for a two tailed z-test are provided in the table (n.s.: not significant). Note that Trpv1 expressing neurons are significantly over-represented in the Dropseq data and Mrgprd cells are significantly under-represented both when STAMPs and clusters are analyzed. (b) Quantitation of positive cells in trigeminal sections probed by double label ISH with marker genes and Trpv1 compared to Dropseq positives by STAMP and cluster. Because Scn9a, S100b and Piezo2 probes all had the same RNAscope label, we compared each individually with Trpv1. In Dropseq data Trpv1 is over-represented relative to Scn9a, Piezo2 and S100b expressing neurons when compared with ISH data (P < 0.001, χ2 test for the extreme assumptions concerning overlap of expression both for cluster and STAMP analysis).
(DOCX)
