# Supplementary figures and images for: Diversity amongst trigeminal neurons revealed by high throughput single cell sequencing
Source: PLoS One. 2017 Sep 28;12(9):e0185543. doi: 10.1371/journal.pone.0185543 (PMC5619795; doi:10.1371/journal.pone.0185543)

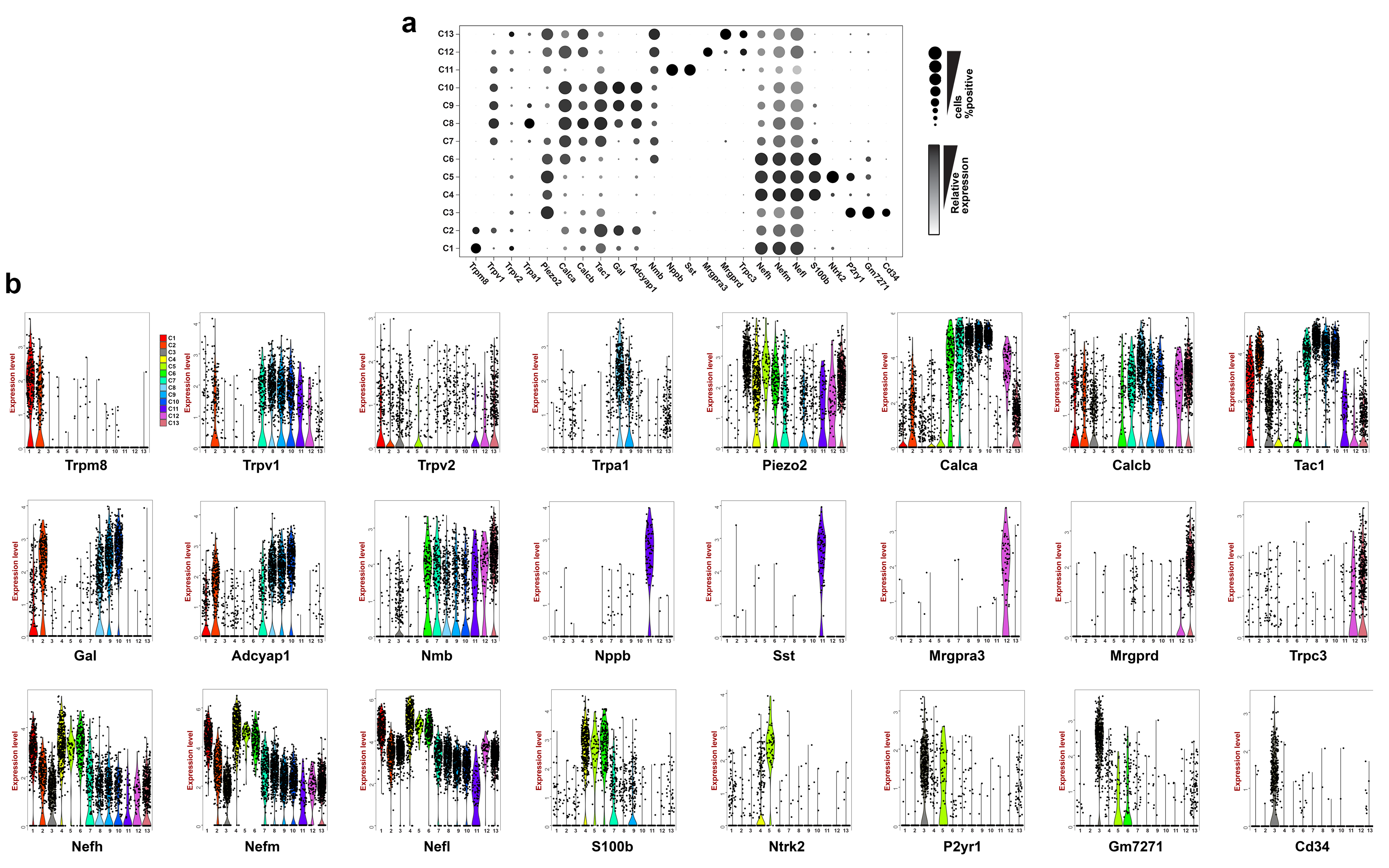

Supplement: S1 Fig — (a) Dot plots depicting transcript representation in STAMPs comprising the 13 clusters C1-13. The diameter of the dot is related to the fraction of STAMPs in that cluster expressing the gene; the shade of the dot reflects the relative level of expression of a transcript in a cluster. (b) Violin plot display of expression data for the same genes: gene expression for individual STAMPs is represented by dots; vertical lines show the maximum expression level in a cluster while colored curves depict the significant expression in individual clusters. The colors are identical to those used in Fig 1c. Note that all these genes exhibit marked differences in expression between the different classes of neurons identified in the clustering analysis. (TIF) [file pone.0185543.s001.tif]

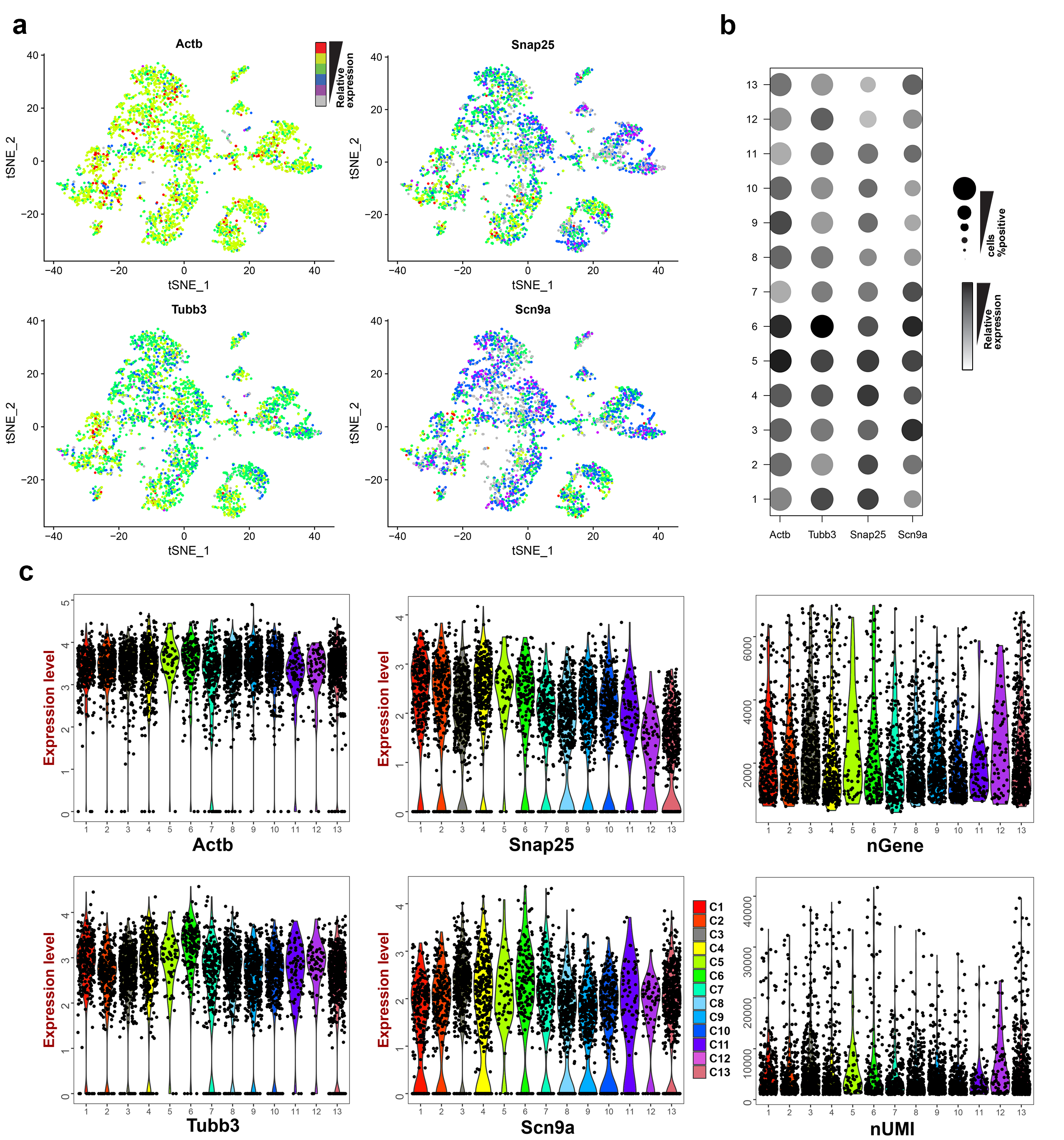

Supplement: S2 Fig — Three different representations of gene expression demonstrate that very highly expressed genes such as Actb or Tubb3 are found in almost every STAMP used in our analysis (98% and 94% respectively). In contrast, other highly expressed genes Snap25 and Scn9a that are expected to be present in all somatosensory neurons are absent from a substantial number of STAMPS as indicated in (a) the tSNE representation, (b) the dot plot and (c) the violin plot; Snap25 and Scn9a are present in 81% and 79% of STAMPs. Note that the dropout of Snap25 and Scn9a is cluster dependent and the cluster specific patterns of expression for these two genes do not match as can be seen in (a) the tSNE representation. Dot plot analysis (b) shows that dropout is greater (smaller dots) for clusters where average relative expression is lower (paler shade). This is also apparent from the violin plot (c) where the extent of the null population is related to the size of the lower bulge. Note neither the number of genes (nGene) nor the number of UMIs (nUMI) is dramatically different between clusters. (TIF) [file pone.0185543.s002.tif]

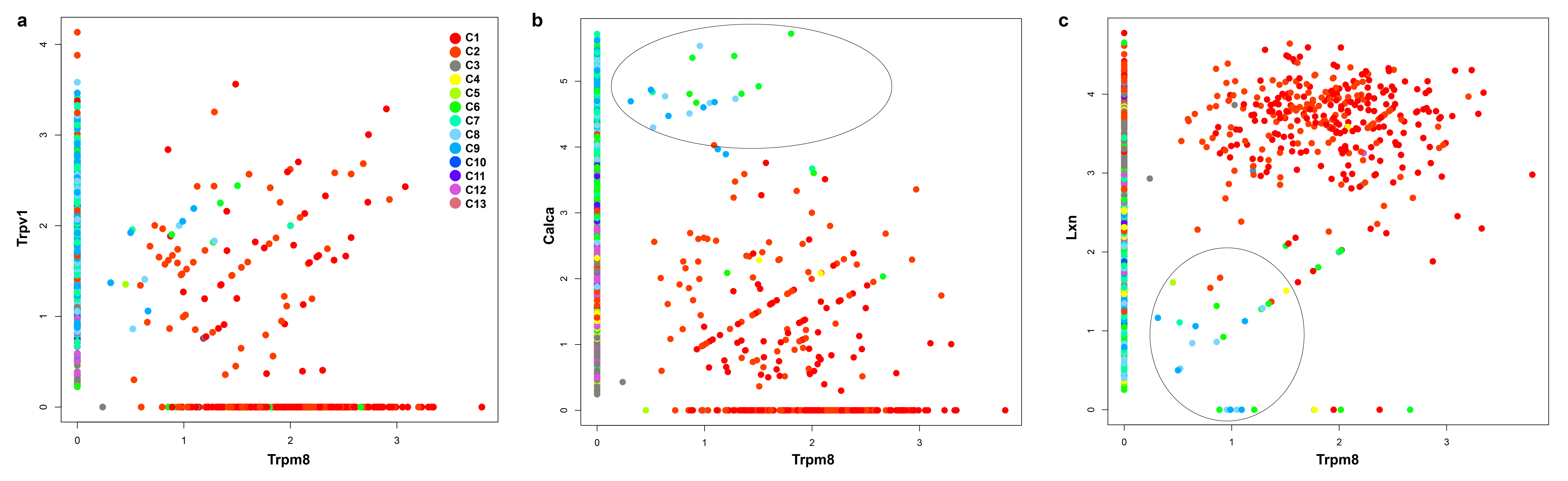

Supplement: S3 Fig — Shown in each graph are the relative expression of a markers and Trpm8 within STAMPs that are positive for either gene; the points represent a single STAMP and are color coded according to cluster using the colors shown in Fig 1c. (a) STAMPs co-expressing Trpm8 and Trpv1 are mainly clustered in C1 (red) and C2 (orange) with only a few STAMPs from the main Trpv1 clusters (blue) being positive for both genes. Most Trpm8 neurons that do not cluster in C1 and C2 (circled, including those that express Trpv1) express high levels of (b) Calca, a marker of peptidergic nociceptors and low levels of (c) Lxn which is prominently expressed in almost all C1 and C2 neurons. (TIF) [file pone.0185543.s003.tif]

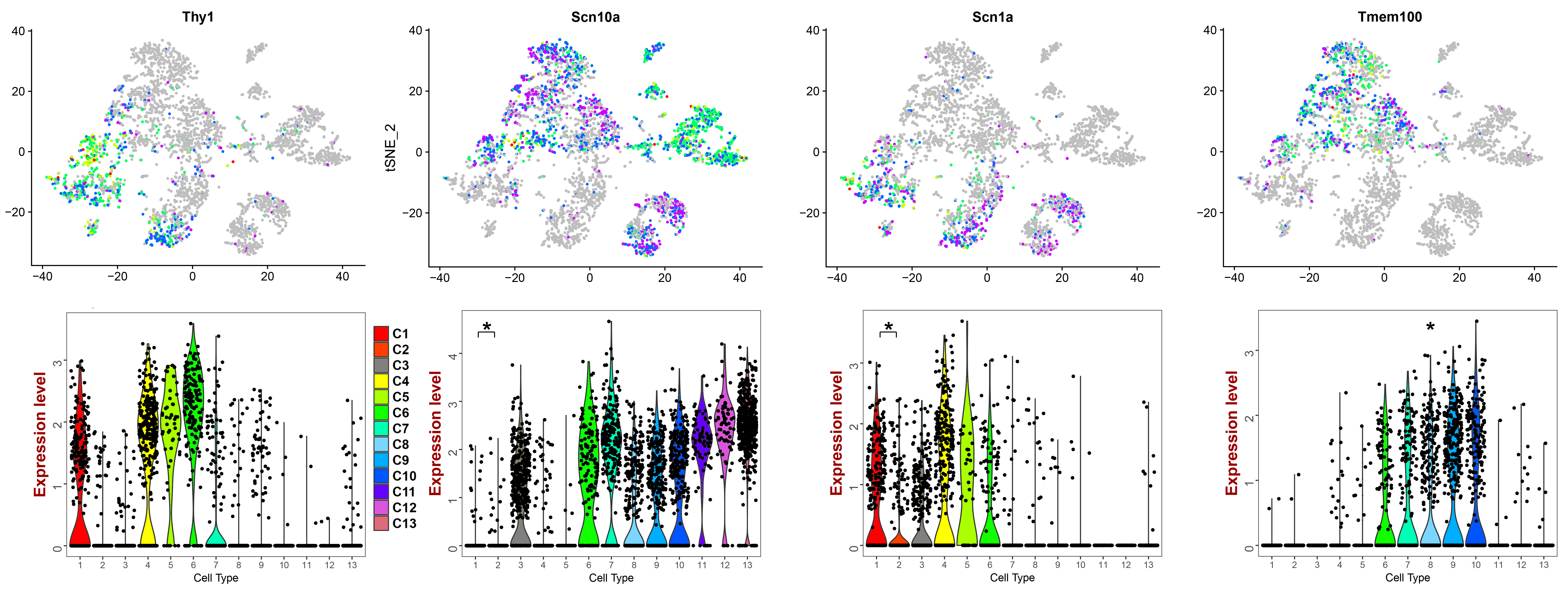

Supplement: S4 Fig — Expression profiles for marker gene Thy1, the functionally important sodium channels Scn10a and Scn1a as well as the transmembrane protein Tmem100 represented in t tSNE analyses (upper panels) and violin plots (lower panels). Thy1 is primarily expressed in neurons that express the highest levels of Nefh, Nefm and Nefl and thus large diameter neurons. Note Scn10a which is required for aversive responses to noxious cold is not expressed in the Trpm8 clusters C1 and C2 (starred). In contrast, Scn1a is prominently expressed in these neurons. Tmem100 was recently reported to mediate interactions of Trpv1 and Trpa1 that are important for pain sensation. However, whereas Trpv1 and Trpa1 co-expression is strongest in C8 (starred), Tmem100 is prominently expressed in other clusters including C6, which are a group of large (Thy1 high), Piezo2 positive neurons likely involved in mechanosensation that generally do not express either Trpv1 or Trpa1 but do express Calca (see Fig 8). (TIF) [file pone.0185543.s004.tif]
