# Supplementary material for: Diversity amongst trigeminal neurons revealed by high throughput single cell sequencing
Source: PLoS One. 2017 Sep 28;12(9):e0185543. doi: 10.1371/journal.pone.0185543 (PMC5619795; doi:10.1371/journal.pone.0185543)
Supplement: S2 Table — Genes are shown in the order displayed in the heatmap (Fig 2); the genes and order were chosen semi-empirically from top markers to highlight the similarities and differences between clusters. (DOCX) [file pone.0185543.s006.docx]

**S2 Table. List of genes used for heatmap shown in Fig. 2**

| Plcd4 | Hpca | Chchd10 | Lxn | Rarres1 | Cdh8 | Trpm8 |
| --- | --- | --- | --- | --- | --- | --- |
| Cpne9 | Gpr26 | Foxp2 | Ccnd2 | Fam184b | Ctxn3 | Omg |
| Ociad2 | Thy1 | Kcnk1 | Cald1 | Gfra3 | Gal | Fam89a |
| Rnase4 | Fxyd6 | Ndrg1 | P2ry1 | Fam19a4 | Cd34 | Gm7271 |
| Cdh9 | Sema3e | Pou4f2 | Nrp1 | Adamts9 | Piezo2 | Kcnd3 |
| S100b | Thy1 | Kcna1 | Cpne6 | Scn1a | Lgi3 | Sfrp1 |
| Syt2 | Atp2b2 | Vsnl1 | Gda | Mgst3 | Sparcl1 | Cygb |
| Cdh13 | Nsg1 | Mef2c | Ntrk2 | Cadps2 | Baiap2l1 | Tmem72 |
| Lgi2 | Lypd1 | Pou4f2 | Ntrk1 | Chp2 | Trappc3l | S100a16 |
| Calca | Tac1 | Tmem100 | Trpv1 | Gal | Gpx3 | Adcyap1 |
| Gfra3 | S1pr3 | Zeb2 | Kit | Cyp1b1 | Ctxn3 | Kcnk18 |
| Aprt | Kitl | Acpp | Ptger1 | Il4ra | Gpr83 | Mfap5 |
| Gabra3 | Lpar1 | Trpa1 | Ccl21a | Trappc3l | Sh3d19 | Resp18 |
| Sstr2 | Gpr64 | Dcdc2a | Gsta4 | Nppb | Sst | Htr1f |
| Npy2r | Tesc | Ildr2 | Il31ra | Cysltr2 | Laptm4b | S1pr1 |
| Etv1 | Mrgpra3 | Nrp1 | Nxn | 4930529M08Rik | Rgs2 | Pde1c |
| Mrgprd | Trpc3 | Lpar3 | Moxd1 | Prkcq | Agtr1a | Kcnab1 |
| Nnat | Gna14 |  |  |  |  |  |

Genes are shown in the order displayed in the heatmap; the genes and order were chosen semi-empirically from top markers to highlight the similarities and differences between clusters.
