# Supplementary material for: Diversity amongst trigeminal neurons revealed by high throughput single cell sequencing
Source: PLoS One. 2017 Sep 28;12(9):e0185543. doi: 10.1371/journal.pone.0185543 (PMC5619795; doi:10.1371/journal.pone.0185543)
Supplement: S3 Table — Counts of positive cells using markers for trigeminal clusters, pan-neuronal genes and groups of neurons from multi-label ISH were compared with counts of positive cells in the Dropseq dataset. (a) Quantitation of positive cells in sections of the trigeminal ganglion probed by ISH with the pan-neuronal marker Scn9a in combination with one or two other markers of trigeminal neurons (see Methods). Positive STAMPs and STAMPs in the major clusters positive for a marker are also shown. We calculated z-scores between normally distributed proportions to determine whether differences between ISH and Dropseq data are significant; P values for a two tailed z-test are provided in the table (n.s.: not significant). Note that Trpv1 expressing neurons are significantly over-represented in the Dropseq data and Mrgprd cells are significantly under-represented both when STAMPs and clusters are analyzed. (b) Quantitation of positive cells in trigeminal sections probed by double label ISH with marker genes and Trpv1 compared to Dropseq positives by STAMP and cluster. Because Scn9a, S100b and Piezo2 probes all had the same RNAscope label, we compared each individually with Trpv1. In Dropseq data Trpv1 is over-represented relative to Scn9a, Piezo2 and S100b expressing neurons when compared with ISH data (P < 0.001, χ2 test for the extreme assumptions concerning overlap of expression both for cluster and STAMP analysis). (DOCX) [file pone.0185543.s007.docx]

**S3 Table. ISH demonstrates biases in Dropseq representation of different neural classes**

**a**

|  | Cells positive for gene | | | Total Neurons | | % positive neurons | | | P-value | |
| --- | --- | --- | --- | --- | --- | --- | --- | --- | --- | --- |
| Gene | ISH | Dropseq by | | ISH (Scn9a) | Dropseq | **ISH** | Dropseq by | | ISH vs STAMPs | ISH vs Clusters |
|  |  | STAMP | Clusters |  |  |  | STAMP | Clusters |  |  |
| Trpv1 | 1000 | 1247 | 1461 | 3668 | 3580 | **27** | 35 | 41 | 1.8x10^-13^ | 2.5x10^-36^ |
| Trpm8 | 561 | 383 | 526 | 3668 | 3580 | **15** | 11 | 15 | 4.2x10^-7^ | n.s. |
| Nppb | 14 | 104 | 79 | 503 | 3580 | **3** | 3 | 2 | n.s. | n.s. |
| Cd34 | 84 | 313 | 440 | 503 | 3580 | **17** | 9 | 12 | 2.1x10^-8^ | 1.6x10^-3^ |
| Mrgprd | 1276 | 517 | 511 | 3899 | 3580 | **33** | 14 | 14 | 1.5x10^-82^ | 1.5x10^-82^ |

**b**

|  | Cells positive for gene | | | Positives for TrpV1 | | | Ratio gene/Trpv1 | | |
| --- | --- | --- | --- | --- | --- | --- | --- | --- | --- |
| Gene | ISH | Dropseq by | | ISH | Dropseq by | | **ISH** | Dropseq by | |
|  |  | STAMP | Clusters |  | STAMP | Clusters |  | STAMP | Clusters |
| Scn9a | 3668 | 2834 | 3580 | 1000 | 1247 | 1461 | **3.7** | 2.3 | 2.5 |
| S100b | 1353 | 939 | 786 | 499 | 1247 | 1461 | **2.7** | 0.75 | 0.54 |
| Piezo2 | 1117 | 1789 | 1883 | 453 | 1247 | 1461 | **2.5** | 1.4 | 1.3 |

Counts of positive cells using markers for trigeminal clusters, pan-neuronal genes and groups of neurons from multi-label ISH were compared with counts of positive cells in the Dropseq dataset. (a) Quantitation of positive cells in sections of the trigeminal ganglion probed by ISH with the pan-neuronal marker Scn9a in combination with one or two other markers of trigeminal neurons (see Methods). Positive STAMPs and STAMPs in the major clusters positive for a marker are also shown. We calculated z-scores between normally distributed proportions to determine whether differences between ISH and Dropseq data are significant; p-values for a two tailed z-test are provided in the table (n.s.: not significant). Note that Trpv1 expressing neurons are significantly over-represented in the Dropseq data and Mrgprd cells are significantly under-represented both when STAMPs and clusters are analyzed. (b) Quantitation of positive cells in trigeminal sections probed by double label ISH with marker genes and Trpv1 compared to Dropseq positives by STAMP and cluster. Because Scn9a, S100b and Piezo2 probes all had the same RNAscope label, we compared each individually with Trpv1. In Dropseq data Trpv1 is over-represented relative to Scn9a, Piezo2 and S100b expressing neurons when compared with ISH data (P < 0.001, χ2 test for the extreme assumptions concerning overlap of expression both for cluster and STAMP analysis).
